# Targeting TKI-Activated NFKB2-MIF/CXCLs-CXCR2 Signaling Pathways in FLT3 Mutated Acute Myeloid Leukemia Reduced Blast Viability

**DOI:** 10.3390/biomedicines10051038

**Published:** 2022-04-30

**Authors:** Huynh Cao, Verena Tadros, Benjamin Hiramoto, Kevin Leeper, Christopher Hino, Jeffrey Xiao, Bryan Pham, Do Hyun Kim, Mark E. Reeves, Chien-Shing Chen, Jiang F. Zhong, Ke K. Zhang, Linglin Xie, Samiksha Wasnik, David J. Baylink, Yi Xu

**Affiliations:** 1 Division of Hematology and Oncology, Department of Medicine, Loma Linda University, Loma Linda, CA 92354, USA; hcao@llu.edu (H.C.); chino@llu.edu (C.H.); bpham@llu.edu (B.P.); mereeves@llu.edu (M.E.R.); cschen@llu.edu (C.-S.C.); 2 Loma Linda University Cancer Center, Loma Linda, CA 92354, USA; 3 Division of Regenerative Medicine, Department of Medicine, Loma Linda University, Loma Linda, CA 92354, USA; vtadros@students.llu.edu (V.T.); bhiramoto@students.llu.edu (B.H.); kcleeper1@students.llu.edu (K.L.); jeffreyxiao77@gmail.com (J.X.); dohyun.k4@gmail.com (D.H.K.); swasnik@llu.edu (S.W.); dbaylink@llu.edu (D.J.B.); 4 Department of Basic Sciences, Loma Linda University, Loma Linda, CA 92354, USA; jzhong@llu.edu; 5 Department of Nutrition, Texas A&M University, College Station, TX 77030, USA; kzhang@tamu.edu (K.K.Z.); linglin.xie@ag.tamu.edu (L.X.); 6 Center for Epigenetics & Disease Prevention, Institute of Biosciences & Technology, College of Medicine, Texas A&M University, Houston, TX 77030, USA

**Keywords:** AML, FLT3, NFKB2, CXCR2, MIF, gilteritinib, CD44, cytokine, tyrosine kinase inhibitor, compensation

## Abstract

Disease relapse is a common cause of treatment failure in FMS-like tyrosine kinase 3 (FLT3) mutated acute myeloid leukemia (AML). In this study, to identify therapeutic targets responsible for the survival and proliferation of leukemic cells (blasts) with FLT3 mutations after gilteritinib (GILT, a 2nd generation tyrosine kinase inhibitor (TKI)) treatment, we performed proteomic screening of cytokine release and in vitro/ex vivo studies to investigate their associated signaling pathways and transcriptional regulation. Here, we report that macrophage migration inhibition factor (MIF) was significantly increased in the supernatant of GILT-treated blasts when compared to untreated controls. Additionally, the GILT-treated blasts that survived were found to exhibit higher expressions of the *CXCR2* gene and protein, a common receptor for MIF and pro-inflammatory cytokines. The supplementation of exogenous MIF to GILT-treated blasts revealed a group of CD44High+ cells that might be responsible for the relapse. Furthermore, we identified the highly activated non-classical NFKB2 pathway after GILT-treatment. The siRNA transient knockdown of *NFKB2* significantly reduced the gene expressions of *MIF*, *CXCR2*, and *CXCL5*. Finally, treatments of AML patient samples ex vivo demonstrated that the combination of a pharmaceutical inhibitor of the NFKB family and GILT can effectively suppress primary blasts’ secretion of tumor-promoting cytokines, such as CXCL1/5/8. In summary, we provide the first evidence that targeting treatment-activated compensatory pathways, such as the NFKB2-MIF/CXCLs-CXCR2 axis could be a novel therapeutic strategy to overcome TKI-resistance and effectively treat AML patients with FLT3 mutations.

## 1. Introduction

Despite significant advancements in our understanding of AML, the relapsed and refractory disease remains a major unmet challenge and the primary reason for the poor overall survival seen in AML [1,2]. Approximately 50% of patients will develop relapsed disease following induction chemotherapy, resulting in a dismal 5-year overall survival rate of 29.5% (NCI SEER). FLT3 inhibitors have been studied for the treatment of high-risk patients with FLT3-mutated relapsed/refractory AML (FLT3mut AML) [3]. While FLT3 inhibitors have shown promising initial responses, complete remission and durable responses are rarely achieved due to the acquisition of drug-resistant clones [4,5]. Primary and secondary resistance to TKIs that developed during the treatment course suggests that alternative signaling pathways may be responsible for the survival of relapsed/refractory blasts [6]. Thus, it is critical to (1) Gain a better understanding of the fundamental intra/extracellular changes of the AML blasts after the treatment; (2) Develop effective therapies targeting the specific pathways underlying AML relapse to overcome treatment-resistance and improve overall survival for AML patients [7].

Inflammation has been found to promote all stages of tumorigenesis in solid tumors [8]. Tumor-promoting inflammatory cytokines, such as chemokine (C-X-C motif) ligands (CXCLs) and their receptors, such as the CXC chemokine receptor (CXCR) family play important roles in mediating the interaction between AML blasts and their microenvironment [9,10]. Both normal and malignant hematopoietic stem cells are known to secrete cytokines and growth factors into their microenvironment for cell survival and proliferation [11,12,13,14]. However, how FLT3mut AML blasts respond to treatment and what kind of autocrine cytokines are released to support their survival and continuous proliferation post-treatment are not fully understood. Filling in such knowledge gaps is crucial for the development of effective targeted therapies to overcome the drug resistance in relapsed/refractory AML with FLT3-mutation. In this study, we designed a series of in vitro and ex vivo experiments to identify cytokines and their transcriptional regulation responsible for the initiation of the survival mechanism and subsequent cell proliferation of FLT3mut AML blasts after TKI treatment. First, human cytokine proteomic arrays were utilized to profile the cytokines changes within cell-free supernatants from GILT-treated MV4-11 cell line. Next, we investigated the correlation of selected TKI-activated cytokines, their signaling pathways and transcriptional regulation to the survival mechanism of TKI-treated blasts. Finally, we explored whether the combination of GILT and multiple inhibitors of receptors and transcription factors controlling the signal transduction of pro-inflammatory cytokines would effectively treat ex vivo primary blasts from FLT3mut AML. We found that TKI-activated NFKB2-MIF/CXCLs-CXCR2 pathways could be a novel target for the treatment of FLT3mut AML. 

## 2. Materials and Methods

The list of reagents including manufacturers and catalogs of antibodies, kits and qPCR primers are found in the supplementary data (Supplementary Tables S1 and S2).

### 2.1. Primary Leukemia Blasts

FLT3mut AML bone marrow (BM) mononuclear cells (BMMNC) (Patients #1-6, Table 1) were obtained from the City of Hope National Medical Center (COHNMC). All donor patients signed an informed consent form. Sample acquisition was approved by the Institutional Review Boards at the LLUMC and the COHNMC in accordance with an assurance filed with and approved by the Department of Health and Human Services, and it (IRB#:58238, 11-10-2021) met all requirements of the Declaration of Helsinki. 

### 2.2. Cell Culture of AML Cells and Treatment of Blasts by TKIs

MV4-11 (ATCC CRL-9591) is a patient-derived AML blast cell line with FLT3-ITD. The FLT3mut AML cells (either MV4-11 or primary AML BMMNC) were cultured in RPMI-1640 medium (Hyclone, Thermo Scientific, Waltham, MA, USA), supplemented with 10% heat-inactivated fetal bovine serum (FBS, HyClone, Citiva, Brooklin, NY, USA) and 1% penicillin/streptomycin. Cells were grown at 37 °C in a humidified atmosphere containing 5% CO_2_. Gilteritinib (GILT) and Quizartinib (QUIZ) are second-generation FLT3 inhibitors [15]. As single agents to treat blasts in vitro, a single dose of 80 nM of GILT or QUIZ was added to 1 ml of 1 × 10^6^ MV4-11 cells for each experimental group in 24 well plates. Three days after the one dose treatment, cells were then collected for qPCR analyses of their gene expressions for *CXCR2*, *CD44*, *CD74*, *NFKB1*, *NFKB2*, etc., some of which were confirmed by the flow cytometry assay of protein expressions. 

### 2.3. In Vitro & Ex Vivo Treatment of MIF and Small Molecular Inhibitors 

The list of recombinant human MIF, inhibitors, abbreviations, manufacturers, and catalog # are found in the supplementary data (Supplementary Table S1). (1) For MIF in vitro experiments, different doses of MIF were added to 1 mL of 1 × 10^6^ MV4-11 cells or RAW264.7 cells for each experimental group in 24-well plates. RAW264.7 is a macrophage cell line (Sigma-Aldrich 91026702-1VL, Burlington, MA, USA). Two days after the one dose treatment, cells were then collected for either analysis by flow cytometry (FC) or qPCR. (2) As combination agents to treat blasts in vitro/ex vivo, one dose of 80 nM GILT with one dose of different inhibitors were added to 1 mL of 1 × 10^6^ MV4-11 cells or 1 mL of 0.5–1 × 10^6^ primary FLT3mut AML BMMNC for each experimental group in 24-well plates. We also performed dose-dependent experiments of CXCR2-I and NFKB-I with or without 80 nM GILT, and the current dose for either 50 µM CXCR2-I or 50 µM NFKB-I is the most effective dose on MV4-11 based on our FC analyses (Figure 4A). Three days after the one dose treatment, cells were then collected for either analysis by FC and/or qPCR.

### 2.4. Combination of GILT and Recombinant Human MIF Protein to Treat MV4-11 In Vitro

(a) Sequential coculture: after 3 days’ treatment of 80 nM GILT, appropriate doses of recombinant human MIF were added into GILT-treated cells for continuous cultures for two days. 

(b) Simultaneous coculture: 80 nM GILT and appropriate doses of MIF were added to the culture medium at the same time in vitro for 3 days, and then another dose of MIF was added for continuous cultures for two days. After 5 days, cells from either sequential or simultaneous cultures were collected for FC assay and qPCR assay. 

### 2.5. Cell Proliferation Assay

BrdU incorporation assay was performed as previously reported [16]. Briefly, the detection of cell proliferation has utilized the incorporation of the thymidine analog BrdU (5-bromo-2′-deoxyuridine, Abcam, Cambridge, UK) during DNA synthesis, followed by detection with an anti-BrdU antibody. Ki-67 presents during all active phases of the cell cycle and is a potential biomarker for the proliferation of AML cells [17]. 

### 2.6. Transfection of siRNA-NFKB2 in MV4-11

A small interfering RNA (siRNA) was used to transiently knockdown *NFKB2* (p100/52) in MV4-11 cells. Cells transfected with scrambled siRNA were used as control (siRNA-NegControl). The cells were grown in 24-well plates and transfected with siTran 2.0 siRNA transfection reagent (Origene Cat# TT320002) using 50 nM NFKB2 (human, ID 4791) 27mer siRNA duplexes (#SR303162, Origene, Rockville, MD, USA). In experiments with GILT treatment, one dose of 80 nM Gilteritinib was added to MV4-11 cells with siRNA-NFKB2 in 24 well plates at the same time. The qPCR experiments, including confirmation of knockdown and gene changes, were performed 2 days after transfection. 

### 2.7. Flow Cytometry (FC)

Cells were harvested and examined for the expression of cell surface biomarkers (CD) and intracellular proteins by multichromatic FC as previously described [18]. About 1 × 10^4^~10^6^ cells in 100 µL FC buffer (PBS containing 1% FBS and 0.05% sodium azide) were stained with various fluorescence-conjugated antibodies specific for the desired cell surface proteins at 4 °C for 30 min. The surface-stained cells were then fixed and permeabilized using the appropriate reagents (e.g., the BD Pharmingen Cytofix/Cytoperm buffer, Franklin Lakes, NJ, USA) and stained with different fluorescence-conjugated antibodies specific for the desired intracellular proteins at 4 °C for 2 h or overnight in the permeabilizing buffer (e.g., the BD Perm/Wash buffer). Concentrations of the Abs were used per the manufacturers’ recommendations. Finally, the cells were washed twice in the permeabilizing buffer and twice in the FC buffer before being analyzed on the BD FACSAria II. Data were analyzed using the FlowJo software (FlowJo^TM^ v10, Ashland, OR, USA).

### 2.8. RNA Isolation and Real-Time Polymerase Chain Reaction (qPCR) Analysis

MV4-11 or primary BMMNC was treated in vitro for appropriate days. Then treated cells were collected for RNA isolation and qPCR analysis as previously described [19]. Total RNA was isolated using the RNeasy Micro Kit (Qiagen, Germantown, MD, USA) according to the manufacturer’s instructions. First-strand cDNA was synthesized using the SuperScript III Reverse Transcriptase (Invitrogen; Life Technologies, Waltham, MA, USA). With an Applied Biosystems 7900HT Real-Time PCR machine, qPCR was performed and analyzed. Primers used in this study are available in Supplementary Table S2. The PCR conditions were 10 minutes at 95 °C followed by 40 cycles of 10 seconds at 95 °C and 15 s at 60 °C. The relative expression level of a gene was determined using the ΔΔ*C*t method and normalized to GAPDH. 

### 2.9. Proteomics and Blotting Analysis

Three days after the one dose treatment, the cell-free supernatants were collected for human cytokine profile assay. The Human XL Cytokine Array Kit (Catalog # ARY022B, R&D, Minneapolis, MN, USA) was used according to the manufacturer’s procedures and exposed to X-ray film (<10 min). Profiles of mean spot pixel density were created using a transmission-mode scanner and Image J analysis software (NIH, Besthesda, MD, USA). 

### 2.10. Imaging Acquisition

Phase-bright images were taken using an Olympus 1X71 inverted microscope and were processed using an Olympus cellSens Dimension 1.15 Imaging Software (Shinjuku-Ku, Tokyo, Japan). 

### 2.11. Statistical Analysis

Statistical significance was assessed by ANOVA or by an independent student “*t*” test for comparison between two or more groups. All values were presented as mean ± SEM. Results were considered significant when the *p*-value was <0.05.

## 3. Results

### 3.1. The Release of MIF Was Significantly Increased by GILT-Treated MV4-11 Cells In Vitro 

**Figure 1:**
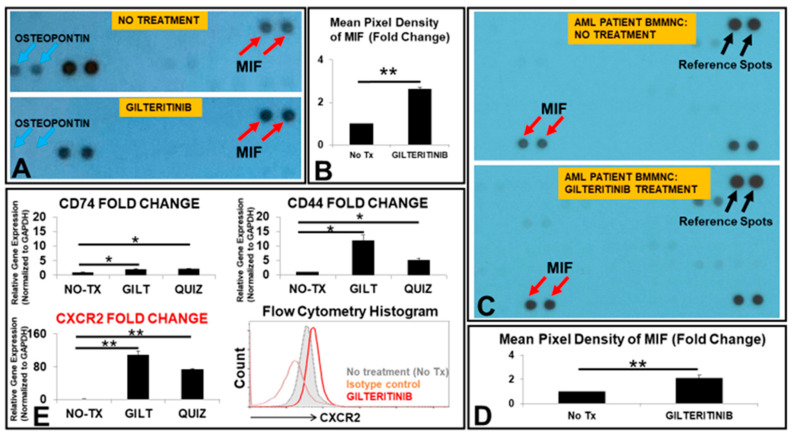
MIF was released more by TKI-treated AML blasts in vitro and ex vivo. (**A**) Image of partial blot films developed for proteomic analyses of cell-free supernatants from 80 nM gilteritinib (GILT)-treated MV4-11 cells. The blue arrows indicate the dots of Osteopontin at the same location in the film. The red arrows indicate the dots of MIF at the same location in the film. Note: Each antibody has two dot spots according to the manufacturer’s specification. (**B**) Cumulative Mean Pixel Densities of MIF (Fold Change); (**C**) Image of partial blot films developed for proteomic analyses of cell-free supernatants from 80 nM GILT-treated primary AML BMMNC cell (Patient #1). The black arrows indicate the reference spots (control dots) from the manufacturer. The red arrows indicate the dots of MIF at the same location in the film. (**D**) Cumulative Mean Pixel Densities of MIF (Fold Change from AML BMMNC, Patient #1); (**E**) 3 days after the treatment of 80 nM gilteritinib (GILT) or quizartinib (QUIZ) in vitro, the cells were collected for RNA isolation and gene expressions were analyzed by qPCR. Data show mRNA expressions of the genes encoding different receptors CD74, CD44 and CXCR2 for MIF; Representative FC histogram plot of CXCR2 expression in GILT-treated MV4-11 cells after 3 days in vitro; * *p* < 0.05, ** *p* < 0.01.

To identify therapeutic targets responsible for the survival and proliferation of TKI-treated FLT3mut AML blasts, we performed proteomic screenings of human cytokines secreted in the cell-free supernatants from GILT-treated MV4-11, a FLT3mut AML cell line. Under the condition without treatment (No Tx), MV4-11 was found to release different cytokines in their supernatant; however, one dose of GILT-treatment significantly reduced the release of a variety of cytokines, such as Osteopontin (Figure 1A), a protein closely associated with adverse prognosis in AML patients [20]. Notably, MIF was upregulated by 2.6-fold in the supernatant of treated MV4-11 blasts when compared to the untreated control (Figure 1A,B). We also examined the release of MIF in GILT-treated bone marrow mononuclear cells (BMMNC) ex vivo. In the GILT-treated FLT3mut AML patient-derived samples (patients #1 and 2, Table 1), MIF release was significantly increased by 2.1-fold in the supernatant when compared to the untreated control (Figure 1C,D). GILT-treatment caused significant cell death among the blasts (~40% reduction in blasts) whereas there was a continuously increased number of blasts in non-treated control groups (~200% increase). Thus, the mean pixel densities of MIF per viable GILT-treated MV4-11 cell could be much higher than the mean pixel densities of MIF per viable non-treated MV4-11 cell (data not shown). Next, to investigate which transmembrane proteins interact with MIF after TKI treatments, we compared gene expressions of all encoded MIF receptors including *CD74*, *CD44*, *CXCR1*, *CXCR2* and *CXCR4* [21]. Our qPCR data showed significantly increased expression of *CXCR2* mRNA (109-fold) in GILT-treated MV4-11 cells, the highest amongst all screened MIF receptors (Figure 1E). Similar expression changes in *CXCR2* and other MIF receptors’ mRNA occurred in quizartinib (QUIZ, another 2nd generation TKI)-treated MV4-11 cells compared to those in control cells (Figure 1E). Consistent with increased gene expression of *CXCR2* after the TKI treatment, CXCR2 protein was also found to be upregulated (3 days after GILT-treatment, flow cytometry histogram, Figure 1E). Notably, more CXCR2 proteins were located intracellularly than on the surface of FLT3mut AML blasts (Supplementary Figure S1A). When we continued the culture of GILT-treated MV4-11 cells by medium change and replating for another week, we found that there was a continuous increase in CXCR2 proteins at both the surface and intracellular locations of survived and recovered blasts (flow cytometry histogram, Supplementary Figure S1B), suggesting its potential role in the relapse of FLT3mut AML. Increased CXCR2 expression was found to be associated with poor prognosis in AML patients [22]. In summary, our data showed that (1) both a FLT3mut AML cell line and primary FLT3mut BMMNC released more MIF after TKI treatment; (2) TKI-treated MV4-11 cells that survived had significantly upregulated gene and protein expressions of CXCR2, a common receptor for MIF and many pro-inflammatory cytokines, which could be a valuable target for immunotherapy to treat FLT3mut AML. 

### 3.2. MIF Promoted the Proliferation of MV4-11 through the Activation of MIF/CXCLs-CXCR2 Signaling Pathways

**Figure 2:**
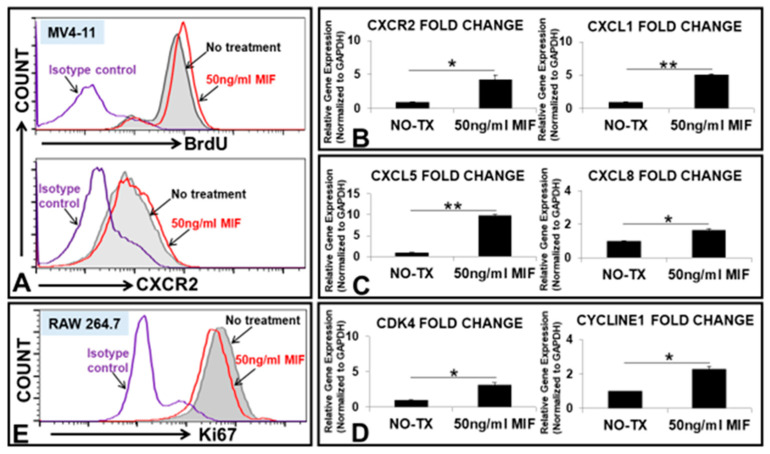
MIF promoted the proliferation of MV4-11 through up-regulating the expressions of CXCR2, cytokines and cell division proteins in vitro. (**A**) Representative flow cytometry (FC) plots of BrdU expression (upper histogram) and CXCR2 expression (lower histogram) in 50 ng/mL MIF-treated MV4-11 cells or non-treatment control (without adding MIF) after 2 days in vitro; (**B**) Data show the change of mRNA expressions of *CXCR2* gene and *CXCL1* chemokine gene in MV4-11 cells at 50 ng/mL MIF in vitro; (**C**) Data show the change of mRNA expressions of *CXCL5* and *CXCL8* chemokine genes in MV4-11 cells at 50 ng/mL MIF in vitro; (**D**) Data show the change of mRNA expressions of *CDK4* and *CYCLIN E1* genes in MV4-11 cells at 50 ng/mL MIF in vitro; (**E**) Representative FC histogram plot of Ki-67 expression in RAW264.7 experimental groups after 2 days in vitro; Red curve indicates the treated group with 50 ng/mL MIF, showing less expression of Ki-67 when compared to the non-treatment control (gray curve); * *p* < 0.05, ** *p* < 0.01.

A significant increase in MIF expression has been related to angiogenesis, cell cycle initiation and tumor metastasis in solid tumors; however, the exact role of MIF in FLT3mut AML is not yet fully understood [23]. To explore the functional role of MIF on FLT3mut AML blasts, we added different doses of recombinant human MIF peptides to the cell cultures of MV4-11. 50 ng/mL MIF promoted MV4-11’s proliferation by displaying greater percentages of BrdU+ and CXCR2+ expressions (flow cytometry histograms, Figure 2A). The qPCR analyses also confirmed that MIF can effectively increase the gene expressions of *CXCR2* mRNA, chemokine (C-X-C motif) ligand *(CXCL) 1*, *CXCL5*, *CXCL8* (*IL8*), *Cyclin-dependent kinase 4 (CDK4)*, and *CYCLIN E1* in MV4-11 cells (Figure 2B–D). To investigate how MIF affects the immune cells in the AML microenvironment, we tested MIF’s effect on macrophages, a common immune cell in the bone marrow. MIF was found to suppress the proliferation of the RAW264.7, a macrophage cell line (Figure 2E). In summary, our data showed that (1) MIF can induce the proliferation of MV4-11; (2) MIF can induce the gene and protein expressions of CXCR2, a shared receptor for a large variety of cytokines; (3) MIF can induce gene expressions of pro-inflammatory cytokines and cell division related regulatory proteins. 

### 3.3. MIF Promoted the Survival of A Group of CD44High+ Cells after the TKI Treatment In Vitro

**Figure 3:**
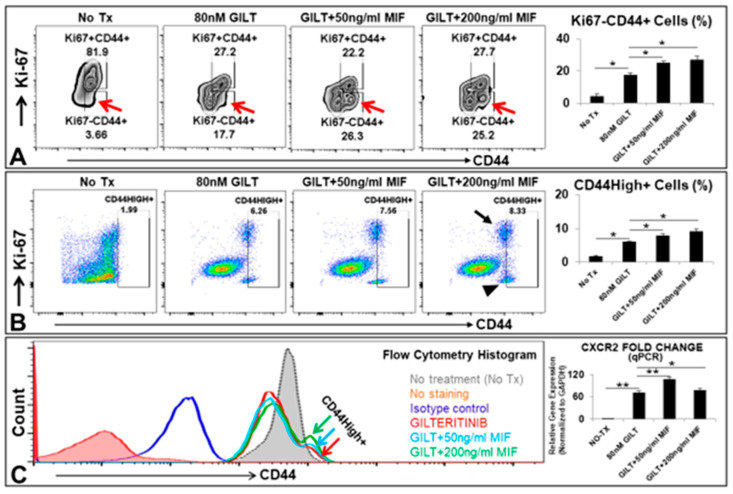
MIF promoted the survival of a group of CD44High+ cells after TKI treatment in vitro. (**A**) Representative FC plots of Ki-67 and CD44 expressions in MV4-11 experimental groups after 5 days’ sequential coculture of 80 nM GILT and appropriate doses of MIF in vitro; Red arrow indicates viable Ki-67-CD44+ cells; Right bar chart: Cumulative FC percentage data of viable Ki-67-CD44+ cells; (**B**) Representative FC plots of Ki-67 and CD44 expressions in MV4-11 experimental groups after 5 days’ simultaneous coculture of 80 nM GILT and appropriate doses of MIF in vitro; Black arrow or arrowhead indicates Ki-67+CD44+ or Ki-67-CD44+ cell population respectively; Right bar chart: Cumulative FC percentage data of viable CD44High+ cells; (**C**) Representative FC histogram plot of CD44 expression in MV4-11 experimental groups after 5 days’ simultaneous coculture in vitro; Color arrows indicate groups treated with 80 nM GILT alone or its combination with different doses of MIF, showing GILT-treated groups with the supplementation of MIF had a group of CD44High+ cells when compared to the non-treatment control or GILT alone; Right bar chart: qPCR Data show the change of mRNA expression of *CXCR2* gene in MV4-11 cells at different doses of MIF combined with 80 nM GILT in vitro; * *p* < 0.05, ** *p* < 0.01.

From our own findings and prior reports of CXCLs-CXCR2 in cancer progression [24], we hypothesized that MIF/CXCLs-CXCR2 pathways could be activated by TKI-treatment in viable blasts to support their survival and continuous proliferation. Accordingly, we performed a serial coculture to examine whether the supplementation of exogenous MIF at early (simultaneous) or late stage (sequential) of TKI treatment could rescue the GILT-treated MV4-11 in vitro (Figure 3). CD44, a multifunctional cell surface adhesion receptor, was identified as a key regulator of quiescent AML leukemic stem cells (LSCs) which are highly microenvironment dependent [25,26]. In the sequential coculture (detailed procedures in the method), we discovered that the supplementation of MIF to GILT-treated MV4-11 cells significantly increased a group of the viable quiescent CD44+ cell population (Ki67-, indicated by red arrows, Figure 3A/plots). The percentage of this CD44+ population was found to significantly increase from 3.66% in untreated control to 17.7% in GILT only, 26.3% in GILT+50 ng/mL MIF and 25.2% in GILT+200 ng/mL MIF (Figure 3A). In the simultaneous coculture, another group of CD44High+ cells was also found which included both Ki67+ and Ki67- subsets (Figure 3B). This group of CD44High+ cells could be visualized in the flow cytometry histogram (Figure 3C). Meanwhile, the qPCR data revealed that the supplementation of MIF to GILT-treated MV4-11 cells significantly increased the gene expression of *CXCR2* (bar chart, Figure 3C). CD44high+ stem-like cells have been previously found to be highly tumorigenic, treatment-resistant and responsible for metastatic progression in breast cancer [27,28,29]. Recently, a combined genetic and functional approach to trace the origins of AML relapse demonstrated that therapy-resistant cells were already present at diagnosis and evolved at relapse from either rare LSCs or blasts with stemness transcriptional signatures [30]. Our current findings suggest that the significantly increased MIF after TKI treatment might support a group of preexisting LSCs-like blasts with inducible CD44high+ expression to survive TKI-treatment and result in the relapse of FLT3mut AML.

### 3.4. Targeting the MIF-CXCR2 Pathway in the Treatment of FLT3mut AML Blasts In Vitro

**Figure 4:**
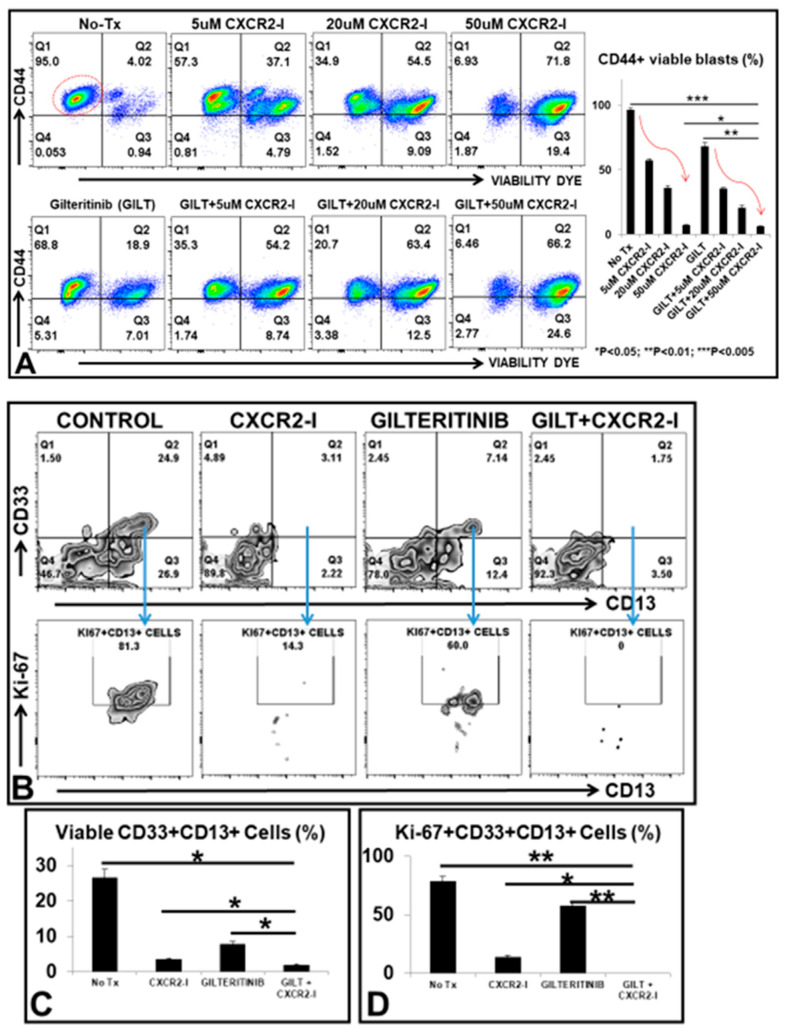
Therapeutic effect of the combination therapy of GILT and CXCR2-I on MV4-11 and primary AML-FLT3 BMMNC in vitro and ex vivo. (**A**) Representative FC plots of viable CD44+ MV4-11 cells (indicated by the red circle in the plot of No-Tx control) in different treatment groups including different doses of CXCR2-I alone or in combination with 80 nM GILT; Right bar chart: Cumulative FC percentage data of viable CD44+ blasts; Red curve arrows indicate the trend of dose-dependent treatment efficacy; (**B**) Representative FC plots of viable CD33+CD13+ primary blasts (Patient #3) in different treatment groups; Blue arrows (Gating) indicate further analyses of Ki-67 expression of these viable CD33+CD13+ primary blasts; (**C**) Cumulative FC percentage data of viable CD33+CD13+ primary blasts; (**D**) Cumulative FC percentage data of viable Ki-67+CD33+CD13+ primary blasts; * *p* < 0.05, ** *p* < 0.01, *** *p* < 0.005.

Next, we investigated whether pharmaceutically targeting the MIF-CXCR2 signaling pathway would effectively improve the therapeutic efficacy of TKI for FLT3mut AML. We explored the combination of GILT with different doses of SB225002, a CXCR2-inhibitor (CXCR2-I) on MV4-11 blasts in vitro. Our flow cytometry data revealed that there was a dose-dependent cytotoxic effect of either CXCR2-I alone (upper plots, Figure 4A) or the combination of GILT plus different doses of CXCR2-I (lower plots, Figure 4A) on MV4-11 in vitro. The percentage of viable CD44+ MV4-11 blasts was significantly reduced from 95% in untreated control cells to 6.46% in GILT+50 µM CXCR2-I, which was superior to 68.8% in GILT only and 6.93% in CXCR2-I only (bar chart, Figure 4A). We also examined the anti-leukemic effect of combining GILT with CXCR2-I on primary BMMNC ex vivo. Our data again showed that 80 nM GILT + 50 uM CXCR2-I significantly reduced the percentage of viable CD33+CD13+ primary blasts (1.75% versus 7.14% in GILT only, 3.11% in CXCR2-I only, and 24.9% in untreated control, Figure 4B/upper plots), and Ki-67+CD13+ proliferating blasts (0% versus 60% in GILT only, 14.3% in CXCR2-I only, and 81.3% in untreated control, Figure 4B/lower plots, Figure 4C/D and Patient #2 and #3, Table 1). However, two patients’ BMMNC specimens did not respond to the combination treatment with the same doses of GILT and CXCR2-I (data not shown, Patients # 1 and 4, Table 1). Our data showing varying responses of primary FLT3mut AML blasts to CXCR2-I is consistent with a previous report that inhibition of the CXCL8-CXCR2 axis moderately reduced the clone proliferation of MDS/AML cell lines and patient samples [31]. One plausible explanation for lacking stable efficacy of using CXCR2-I could be that CXCL8 induced a quick internalization of CXCR2 and delayed recycling back to the cell membrane as previously reported [32,33]. Furthermore, the intracellular CXCR2 (Supplementary Figure S1A) has been shown to activate the NFKB pathway in solid tumors [24], an action that releases different tumor-promoting cytokines to support the surviving blasts through bypassing the targeted therapy (indicated by a red dash arrow in schematic diagram of last figure). 

### 3.5. NFKB2 Could Activate MIF/CXCLs-CXCR2 Pathways in TKI-Treated Blasts In Vitro

**Figure 5:**
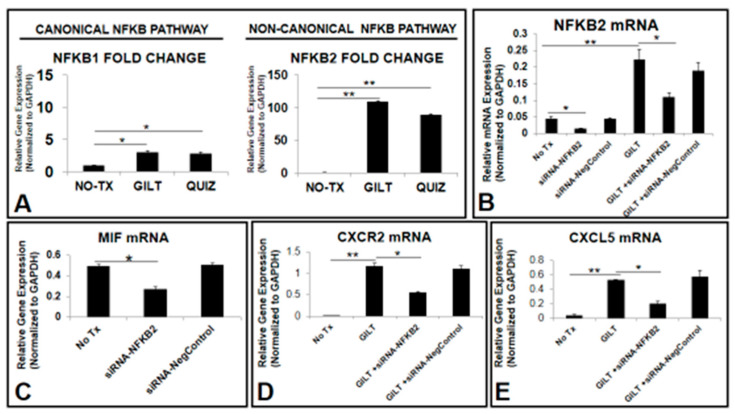
TKI-activated NFKB2 (P100/P52) pathway and siRNA knockdown of *NFKB2* in vitro. (**A**) Data show mRNA expressions of canonical (*NFKB1*) and non-canonical (*NFKB2*) pathways after 80 nM GILT or 80 nM QUIZ treatment; (**B**) 2 days after the siRNA-NFKB2 treatment (50 nM) with or without 80 nM GILT in vitro, the cells were collected for RNA isolation and gene expressions were analyzed by qPCR. Data show mRNA expressions of *NFKB2*; (*N* = 3) (**C**–**E**) Data show mRNA expressions of *MIF*, *CXCR2* and *CXCL5* genes after transiently knocking down *NFKB2* in MV4-11; * *p* < 0.05, ** *p* < 0.01.

Next, we attempted to identify the transcription factor controlling gene expressions of cytokines and their receptors in surviving TKI-treated blasts. Nuclear factor kappa-light-chain enhancer of activated B cells (NFKB), a family of transcription factors including NFKB1 (p50–p65, canonical) and NFKB2 (p52-RelB, non-canonical), is known to regulate the expression of different tumor-promoting cytokines including MIF and their receptors [34,35,36]. Our qPCR data showed significantly increased *NFKB2* mRNA (110-fold) in GILT-treated MV4-11 when compared to the untreated control (NO-TX) (right bar chart, Figure 5A). The increased fold of *NFKB2* was much higher than *NFKB1* (left bar chart, Figure 5A). Similar expression changes in *NFKB1* mRNA and *NFKB2* mRNA occurred in Quiz-treated MV4-11 cells when compared to those in control non-treatment cells (Figure 5A). Next, we performed siRNA knockdown of *NFKB2* in vitro and then examined the genetic changes after the significant reduction of *NFKB2* mRNA (Figure 5B–E). The siRNA knockdown of *NFKB2* significantly reduced the *NFKB2* mRNA by approximately 3-fold (siRNA-NFKB2 versus untreated control, Figure 5B) and by 2-fold in combination with GILT (GILT+ siRNA-NFKB2 versus GILT only, Figure 5B). Transient knockdown of *NFKB2* does not affect the gene expression of *NFKB1* (data not shown). Furthermore, our data showed that transient silencing of *NFKB2* significantly reduced the *MIF* mRNA by 1.5-fold (siRNA-NFKB2 versus untreated control, Figure 5C) and GILT-activated gene expressions of *CXCR2* and *CXCL5* by 2-fold and 2.6-fold, respectively (GILT+siRNA-NFKB2 versus GILT only, Figure 5D,E). Additionally, we generated a new MV4-11 cell line with *NFKB2* knockdown through shRNA-NFKB2. Our preliminary data from analyzing this new cell line is consistent with that of the results from siRNA-NFKB2 experiments (data now shown). In summary, our data suggest that NFKB2-MIF/CXCLs-CXCR2 pathways could play a key role in the survival mechanism of TKI-treated FLT3mut AML blasts.

### 3.6. The Combination of GILT and a NFKB Family Inhibitor (NFKB-I) Significantly Reduced the Viable Primary Blasts from FLT3mut AML Patients Ex Vivo

**Figure 6:**
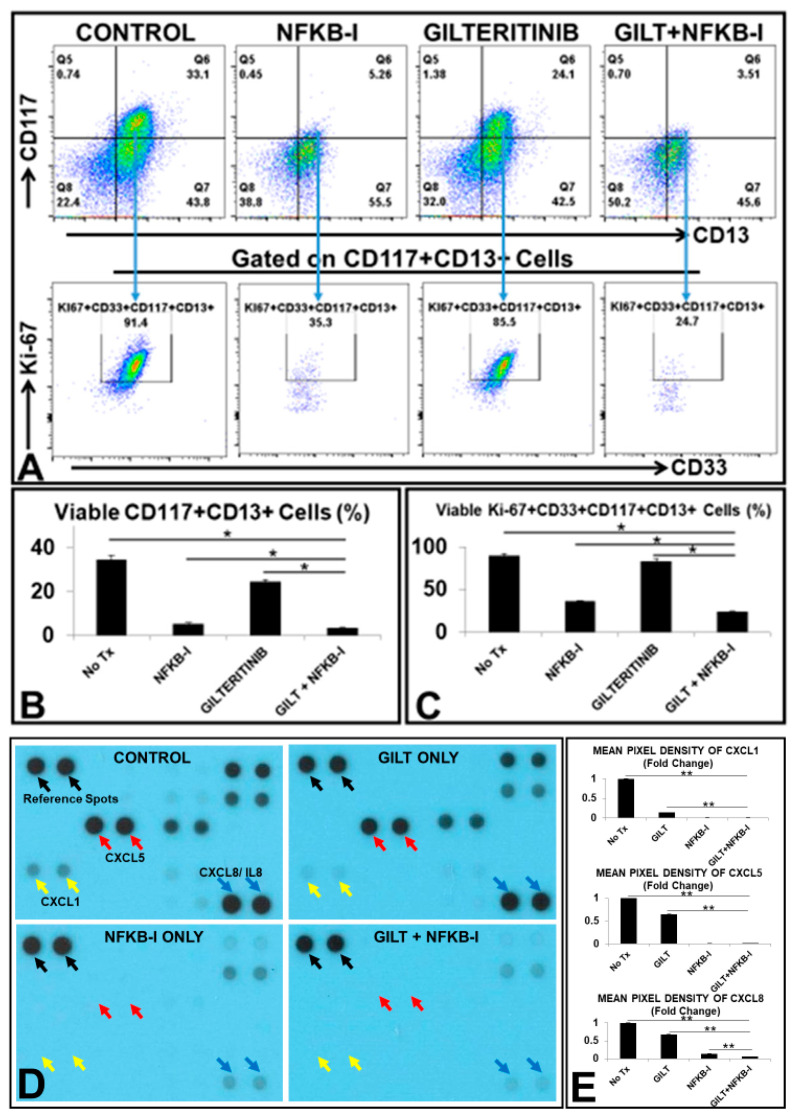
The therapeutic effect of the combination of 80 nM GILT and 50 uM NFKB-I on a newly diagnosed AML-FLT3 BMMNC (Patient #5) ex vivo. (**A**) Representative FC plots of viable CD117+CD13+ primary blasts in different treatment groups; Blue arrows (Gating) indicate further analyses of Ki-67 expression of these viable CD117+CD13+ primary blasts; (**B**) Cumulative FC percentage data of viable CD117+CD13+ primary blasts; (**C**) Cumulative FC percentage data of viable Ki-67+CD33+CD117+CD13+ primary blasts; (**D**) Image of partial blot films developed for proteomic analyses of cell-free supernatants from GILT-treated primary AML BMMNC cells. The black arrows indicate the control dots from the manufacturer. The yellow, red and blue arrows indicate the dots of CXCL1, CXL5 and CXCL8 respectively in the film. (**E**) Cumulative Mean Pixel Densities (Fold Change) of CXCL1, CXCL5, CXCL8. * *p* < 0.05, ** *p* < 0.01.

Finally, we examined the therapeutic efficacy of combining GILT with BAY11-7821 (NFKB-I) on FLT3mut AML blasts. Our data showed that 80 nM GILT + 50 uM NFKB-I significantly reduced the percentage of viable CD117+CD13+ primary blasts (3.51% versus 24.1% in GILT only, 5.26% in NFKB-I only, and 33.1% in untreated control, Figure 6A/upper plots), and Ki-67+CD33+ proliferating blasts (24.7% versus 85.5% in GILT only, 35.3% in NFKB-I only, and 91.4% in untreated control, Figure 6A/lower plots, 6B/C and Patient #5, Table 1). The combination of GILT+ NFKB-I also significantly reduced the amount of key tumor-promoting cytokine proteins, such as CXCL1, CXCL5 and CXCL8 (IL8) in the supernatant of treated BMMNC specimens of FLT3mut AML patients when compared to untreated controls (Figure 6D/blot films and Figure 6E). We also examined the anti-leukemic effect of the combination on a refractory FLT3mut AML patient sample (Patient #6, Table 1). Our data showed that 80 nM GILT + 50 uM NFKB-I not only significantly inhibited the cluster formation in primary BMMNC (Supplementary Figure S2A), but the combination also significantly reduced the viability of CD117+CD13+ blasts (upper plots, Supplementary Figure S2B) and suppressed the proliferation of Ki-67+CD33+CD117+CD13+ primary blasts (lower plots, Supplementary Figure S2B). Our qPCR data confirmed that the combination of GILT and NFKB-I significantly reduced the gene expression of *CYCLIN E1*, a molecule responsible for the S-phase of the cell cycle, and the gene expression of CD44, an adhesion molecule for cluster formation (Supplementary Figure S2C). Lastly, the combinatory treatment effectively eliminated CXCL8 (IL8) in the supernatant of treated FLT3mut AML patient specimens when compared to GILT only (Supplementary Figure S2D). In summary, our ex vivo data suggest that targeting NFKB2-MIF/CXCLs-CXCR2 pathways including GILT+NFKB-I and/or CXCR2-I (disrupting the internalization/ maintenance of NFKB’s activation) might be a novel therapeutic approach to treat both newly diagnosed and refractory FLT3mut AML patients by effectively suppressing the release of tumor-promoting cytokines to overcome TKI-resistance and prevent AML relapse. 

## 4. Discussion

**Figure 7:**
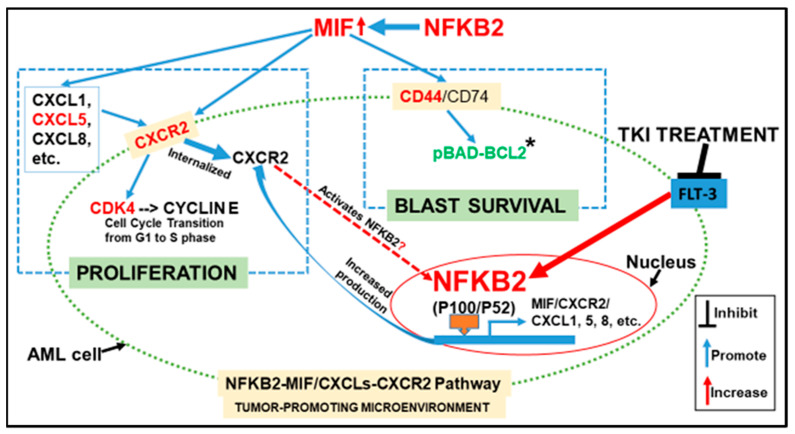
Schematic diagram depicting TKI-activated NFKB2-MIF/CXCLs-CXCR2 compensation pathways responsible for the survival and proliferation of AML blasts. After TKI treatment, cytotoxicity-induced injury signals might directly activate the non-canonical NFKB2 (P100/P52) pathway to release more MIF, CXCL5, CXCL8 and other tumor-promoting cytokines. MIF might act as an autocrine signal to initiate the survival mechanism* through MIF-CD74/CD44 pathways. Meanwhile, MIF-CXCR2, CXCL5-CXCR2 and CXCL8-CXCR2 pathways might be responsible for cell proliferation by activating CDK4/CYCLIN E-based transition from G1 to S phase of the cell cycle progression. * pBAD-BCL2: Phosphorylation of BAD has been restored by PIM family compensation pathways to prevent its binding to BCL-2, allowing blasts to survive the TKI treatment. Red color indicates the key molecules in this study.

In this study, we found in FLT3mut AML that (1) MIF, a tumor-promoting cytokine, was released in greater amounts after TKI-treatment; (2) CXCR2, a receptor for MIF and many pro-inflammatory cytokines was increased by TKI-treatment; (3) NFKB2-MIF/CXCLs-CXCR2 signaling pathways could be responsible for the survival of TKI-treated FLT3mut AML blasts (Figure 7); and (4) The combination of GILT and NFKB-I can effectively treat primary blasts by reducing tumor-promoting cytokines ex vivo. 

AML has the lowest survival rate among the leukemias [1,2]. As a result, there is an urgent need to understand the fundamental intracellular and extracellular mechanisms of treatment resistance and develop effective therapies to overcome the drug resistance and improve the prognosis of AML patients. Tumor-promoting cytokines and their release into the microenvironment are known to play causal roles in the treatment resistance of cancer cells and their regained tumorigenic capabilities in solid tumors [8]. MIF is a pro-inflammatory cytokine that binds to CD74/CD44 receptors, activating pro-survival and proliferative pathways to promote wound healing [37]. MIF/CXCLs-CXCR2 pathways were shown to be up-regulated in solid tumors, and these pathways have been suggested as therapeutic targets to prevent disease progression [38,39,40,41]. The involvement of MIF in FLT3mut AML is not yet fully understood, but there is an ongoing clinical trial (NCT03918655) in the early stages of the investigation. Our current findings showing MIF’s expression in AML cell lines and primary blasts (Figure 1) and MIF-induced expressions of *CXCL1/5/8* and *CXCR2* in MV4-11 cells in vitro (Figure 2) are consistent with a previous report that primary AML blasts constitutively express MIF, stimulating bone marrow mesenchymal cells to release the CXCR2 ligand CXCL8 (IL-8) to sustain blasts’ survival [42]. Our data also suggest that MIF might play a key role in maintaining the routine release of CXCL1/5/8 reported in the supernatants of naïve AML patient cultured cells ex vivo [10]. The regulation of the human *MIF* gene expression was found to involve very complex transcriptional networks, such as Hypoxia-Inducible Factors (HIF families) [35], which are known to confer therapeutic resistance to AML [43]. Thus, it is possible that to survive in the hypoxic condition of AML bone marrow, increased release of MIF promotes a favorable tumor microenvironment by restricting the macrophages and performing an autocrine effect to support the survival and proliferation of blasts through both the MIF-CD74/CD44 pathway and the MIF/CXCLs-CXCR2 pathways in relapsed or refractory FLT3mut AML patients (Figure 7). 

NFKB family transcription factors are known to control the expression of most tumor-promoting cytokines and their receptors [34,44], including the human MIF gene [35]. As a member of “rapid-acting" transcription factors, such as c-Jun, the NFKB family is one of the first responders to harmful cellular stimuli [45] and suppresses apoptosis to promote cancer progression [46]. The NFKB family has been found constitutively activated in AML; however, most studies have focused on the classical pathway of NFKB1/P105/P50 [47]. Evolutionarily, duplicated gene pairs that are “redundant” elements of the compensation system in the cellular system can overlap in function to ensure biological robustness for all living beings [48]. Nevertheless, redundancy might also be an obstacle to cancer treatment [49]. In this study, we found that a non-canonical NFKB2 pathway was more activated than the canonical NFKB1 pathway during TKI treatment (Figure 5A). FLT3-ITD was reported to promote the non-canonical P52/NFKB2 pathway in refractory AML [50]. Additionally, the treatment-activated NFKB2 pathway was found to promote the chemokine release in primary AML cells [51]. Our siRNA knockdown data demonstrated the essential role of NFKB2 in the gene expressions of MIF, pro-survival CXCL cytokines, and their CXCR2 receptor (Figure 5C–E). Thus, we speculate that in survived TKI-treated blasts, the non-canonical NKFB2 pathway is activated to compensate for suppressed NFKB1 and promote the release of pro-survival cytokines, such as MIF/CXCL1/5/8 to initiate the survival and proliferation of TKI-treated blasts (Figure 7). Therapeutic application of inhibition of both canonical and non-canonical NFKB signaling pathways has been in clinical trials to treat solid tumors [52,53]. In this study, our preliminary ex vivo results showed that the combination of GILT and NFKB-I could effectively reduce primary FLT3mut AML blasts by inhibiting their cell cycle initiation, cluster formation and release of pro-survival cytokines. These promising results will need to be confirmed in a larger cohort of FLT3mut AML patients and further evaluated of therapeutic efficacy in AML mouse models in vivo. In summary, we provided the first evidence to support the novel concept that treatment-activated compensation systems could be responsible for AML relapse. Targeting NFKB2-MIF/CXCLs-CXCR2 pathways on top of utilizing TKIs could be a significant step towards preventing the relapse of FLT3-mutated AML.

## Figures and Tables

**Table 1:** Information of AML-FLT3 Patients.

No.	Diagnosis	Age	Sex	Disease Status	Gene Mutations
#1	AML	55	M	Newly diagnosed	1. FLT3: 40% allele frequency (c.2503G>T; p.D835Y) 2. NPM1: SR 0.72 (c.863_864insCTTG; p.W288Cfs*12)
#2	AML	41	M	Relapsed/Refractory	FLT3 Internal Tandem Duplication (ITD): Allele Frequency: (2 separate mutations) 0.25% & 0.52%
#3	AML	69	M	Relapsed/Refractory, On treatment	1. FLT 3 ITD: SR 0.89 (c.1789delins25; p.Y597delins9) (23% allele frequency) 2.RUNX1: 94% allele frequency (c.602G>A; p.R201Q) 3.IDH2: 65% allele frequency (c.419G>A; p.R140Q) 4. DNMT3A: 48% allele frequency (c.2645G>A; p.R882H) 5.SRSF2: 46% allele frequency (c.284_307del24; p.P95_R102del)
#4	AML	53	F	Newly Diagnosed	1. CBFB-MYH11: Allele Frequency 102.3453% 2. FLT3 (c.1793_1794ins21;p.Y597_E598insDDPSLID): Allele Frequency 0.65% 3. KIT (c.1251_1257dekubsGGCA;p.Y418_D419delinsA): Allele Frequency 2%
#5	AML	62	M	Newly diagnosed	1. FLT3-ITD: Level = 0.98
#6	AML	65	F	Refractory	1. FLT 3: ITD SR 0.57 2. PHF6: 41% allele frequency (c.821G>A; p.R274Q) 3. RUNX1: 34% allele frequency (c.494_497dup; p.G168Kfs*46) 4. WT1: 82% allele frequency (c.1140dup; p.S381Vfs*4)

## Data Availability

Not applicable.

## Supplementary Materials

The following supporting information can be downloaded at: www.mdpi.com/article/10.3390/biomedicines10051038/s1, Table S1: List of Reagents used in this study; Table S2: List of Primers (Origene, etc.) used in this study; Figure S1. Increased CXCR2 expression in survived GILT-treated MV4-11; Figure S2. Therapeutic effect of the combination of 80 nM GILT and 50Um NFKB-I on a refractory AML-FLT3 BMMNC (Patient #6) ex vivo.
