# Supplementary material for: Targeting TKI-Activated NFKB2-MIF/CXCLs-CXCR2 Signaling Pathways in FLT3 Mutated Acute Myeloid Leukemia Reduced Blast Viability
Source: Biomedicines. 2022 Apr 30;10(5):1038. doi: 10.3390/biomedicines10051038 (PMC9138861; doi:10.3390/biomedicines10051038)
Supplement: Supplementary file 1 [file biomedicines-10-01038-s001.zip › biomedicines-1685574-supplementary.pdf]

Targeting TKI-Activated NFKB2-MIF/CXCLs-CXCR2 Signaling Pathways in FLT3 mutated Acute Myeloid Leukemia Reduced Blast Viability

Huynh Cao[1, 2,†], Verena Tadros[3,†], Benjamin Hiramoto[3,†], Kevin Leeper[3], Christopher Hino[1], Jeffrey Xiao[3], Bryan Pham[1], Do Hyun Kim[3], Mark E. Reeves[1, 2], Chien-Shing Chen[1, 2], Jiang F. Zhong[4], Ke K. Zhang[5, 6], Linglin Xie[5], Samiksha Wasnik[3], David J. Baylink[3], Yi Xu[1, 2, 3]*

Supplementary Documents

List of Reagents				
**Antibody/Reagents**	Abbreviation/Name in the text	Cat. #	Company	Species Reactivity
**Viability Dye eFluor™ 780**	Viability Dye	65-0865-14	eBioscience	
**CD13-PE-CY7**	CD13	301712	Biolegend	Human
**CD14-APC**	CD14	325608	Biolegend	Human
**CD33-PERCP**	CD33	341640	BD	Human
**CD33-APC**	CD33	303408	Biolegend	Human
**CD33-FITC**	CD33	366620	Biolegend	Human
**CD34-PE**	CD34	348057	BD Biosciences	Human
**CD44-FITC**	CD44	338804	Biolegend	Human
**CD44-PE-Cy7**	CD44	338816	Biolegend	Human
**CXCR2-PE**	CXCR2	320705	Biolegend	Human
**CXCR2-APC**	CXCR2	320710	Biolegend	Human
**CXCR2**	CXCR2	20634-1-AP	Ptglab	Human
**CD117-PERCP**	CD117	313214	Biolegend	Human
**Ki-67-PE**	Ki-67	350504	Biolegend	Human
**Ki-67-APC**	Ki-67	350514	Biolegend	Human
**Ki-67-PERCP**	Ki-67	350519	Biolegend	Human
**BrdU-PERCP**	BrdU	364109	Biolegend	
**BrdU (5-bromo-2'-deoxyuridine)**	BrdU	142567	Abcam	
**IgG1, κ Isotype Ctrl- FITC**		400107	Biolegend	
**IgG1, κ Isotype Ctrl- PE**		400111	Biolegend	

**IgG1, κ Isotype Ctrl- APC**		400121	Biolegend	
**IgG1, κ Isotype Ctrl- PERCP**		400147	Biolegend	
**Gilteritinib (ASP2215)**	GILT	S7754	SELLECKCHEM	
**Quizartinib**	QUIZ	A10027	ADOOQ	
**SB225002**	CXCR2-I	HY-16711	MedChemExpress	
**BAY11-7082 (BAY11-7821)**	NFKB-I	S2913	SELLECKCHEM	
**Recombinant Human MIF**	MIF	300-69	Peprotech	Human
**Human XL Cytokine Array Kit**	Cytokine Array	ARY002B	R&D SYSTEMS	Human
**siRNA Oligo NFKB2 p100/ p52**	siRNA-NFKB2	SR303162	Origene	Human

Supplementary Table S1: List of Reagents used in this study.

#	**Name (HUMAN)**	**Forward Sequence**	**Reverse Sequence**
1	***CD44***	CCAGAAGGAACAGTGGTTTGGC	ACTGTCCTCTGGGCTTGGTGTT
2	***CD74***	AAGCCTGTGAGCAAGATGCGCA	AGCAGGTGCATCACATGGTCCT
3	***CXCR2***	TCCGTCACTGATGTCTACCTGC	TCCTTCAGGAGTGAGACCACCT
4	***CXCL1***	AGCTTGCCTCAATCCTGCATCC	TCCTTCAGGAACAGCCACCAGT
5	***CXCL5***	CAGACCACGCAAGGAGTTCATC	TTCCTTCCCGTTCTTCAGGGAG
6	***CXCL8 (IL8)***	GAGAGTGATTGAGAGTGGACCAC	CACAACCCTCTGCACCCAGTTT
7	***NFKB1***	GCAGCACTACTTCTTGACCACC	TCTGCTCCTGAGCATTGACGTC
8	***NFKB2***	GGCAGACCAGTGTCATTGAGCA	CAGCAGAAAGCTCACCACACTC
9	***MIF***	AGAACCGCTCCTACAGCAAGCT	GGAGTTGTTCCAGCCCACATTG
10	***CYCLIN E1***	TGTGTCCTGGATGTTGACTGCC	CTCTATGTCGCACCACTGATACC
11	***CDK4***	CCATCAGCACAGTTCGTGAGGT	TCAGTTCGGGATGTGGCACAGA

Supplementary Table S2: List of Primers (Origene, etc.) used in this study.

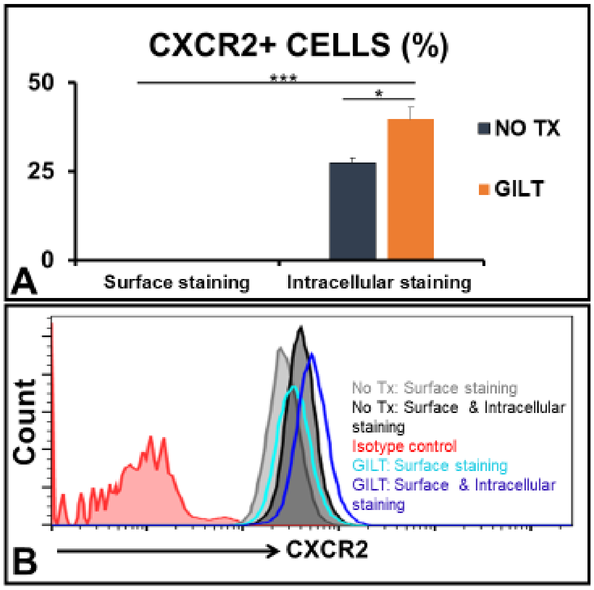

Supplementary Figure S1. Increased CXCR2 expression in survived GILT-treated MV4-11 cells *in vitro*.

A) Cumulative FC percentage data of viable CXCR2+ MV4-11 cells after 3 days' GILT-treatment, which were analyzed by either surface staining or intracellular staining;

B) Representative FC histogram plot of CXCR2 expressions in viable GILT-treated MV4-11 cells *in vitro*;

Briefly, MV4-11 cells were treated with 80nM GILT or without GILT (No Tx) for three days, and then underwent medium change to remove GILT and continuous culture for another week to let survived GILT-treated cells recover. Then GILT-treated cells were collected for FC staining including surface staining and/or intracellular staining to detect CXCR2 expressions. Color curves indicate different staining methods and show increased expressions of CXCR2 in viable GILT-treated MV4-11 cells (light or dark blue curves) when compared to the non-treatment controls (gray or black curves);

*P<0.05, ***P<0.005.

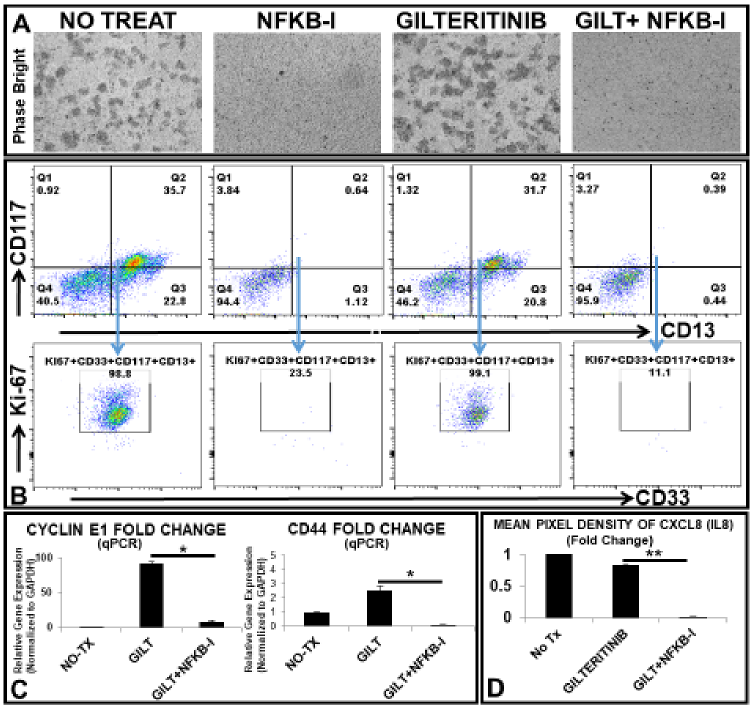

Supplementary Figure S2. Therapeutic effect of the combination of 80nM GILT and 50uM NFKB-I on a refractory AML-FLT3 BMMNC (Patient #6) *ex vivo*.

A) Representative phase-bright images showing tumorigenic cluster formation in different experimental groups 3 days after the treatment of different TKI drugs *ex vivo*;

B) Representative FC plots of viable CD117+CD13+ primary blasts in different treatment groups; Blue arrows (Gating) indicate further analyses of Ki-67 expression of these viable CD117+CD13+ primary blasts;

C) 3 days after the treatment *ex vivo*, the cells were collected for RNA isolation and gene expressions were analyzed by qPCR. Data show mRNA expressions of the genes of *CYCLIN E1* and *CD44*;

D) Cumulative Mean Pixel Densities (Fold Change) of CXCL8, which were acquired from proteome analyses of cell-free supernatants from treated groups of primary AML BMMNC cells. *P<0.05, **P<0.01.
